# More than Fast Food: Development of a Story Map to Compare Adolescent Perceptions and Observations of Their Food Environments and Related Food Behaviors

**DOI:** 10.3390/ijerph16010076

**Published:** 2018-12-28

**Authors:** Kristin A. Riggsbee, Jonathon Riggsbee, Melissa J. Vilaro, Lauren Moret, Marsha Spence, Elizabeth Anderson Steeves, Wenjun Zhou, Melissa D. Olfert, Lisa Franzen-Castle, Tanya Horacek, Elizabeth Hall, Sarah Colby

**Affiliations:** 1 Department of Nutrition, University of Tennessee, Knoxville, TN 37996, USA; kristin.riggsbee@gmail.com (K.A.R.); mspence@utk.edu (M.S.); eander24@utk.edu (E.A.S.); ehall31@vols.utk.edu (E.H.); 2 Blount County GIS Group, Maryville, TN 37801, USA; riggsbeejm@gmail.com; 3 Department of Food Science and Human Nutrition, University of Florida, Gainesville, FL 32612, USA; mgraveley@ufl.edu; 4 Department of Educational Psychology and Counseling, University of Tennessee, Knoxville, TN 37996, USA; lmoret@utk.edu; 5 Department of Business Analytics and Statistics, University of Tennessee, Knoxville, TN 37996, USA; wzhou7@utk.edu; 6 Division of Animal & Nutritional Sciences, School of Agriculture, West Virginia University, Morgantown, WV 26506, USA; Melissa.Olfert@mail.wvu.edu; 7 Department of Nutrition and Health Sciences, University of Nebraska-Lincoln, Lincoln, NE 68588, USA; lfranzen2@unl.edu; 8 Department of Public Health Food Studies and Nutrition, Syracuse University, Syracuse, NY 13244, USA; thoracek@syr.edu

**Keywords:** adolescent, food environment, mapping, critical GIS

## Abstract

The purpose of this convergent, multiphase, mixed methods study was to better understand the perceptions of adolescents’ food environments and related food behaviors using grounded visualization and story mapping. Adolescents from one high school (13–16 years) in the southeastern United States were evaluated via data from health behavior surveys (*n* = 75), school environment maps, focus groups (*n* = 5 groups), and Photovoice (*n* = 6) from October 2016 to April 2017. Data from each phase were integrated using grounded visualization and new themes were identified (*n* = 7). A story map using ArcGIS Online was developed from data integration, depicting the newly identified themes. Participants failed to meet national recommendations for fruit and vegetable intake (2.71 cups). Focus group and Photovoice findings indicated the need for convenience food items in all environments. The story map is an online, interactive dissemination of information, with five maps, embedded quotes from focus groups, narrative passages with data interpretation, pictures to highlight themes, and a comparison of the participants’ food environments. Story mapping and qualitative geographic information systems (GIS) approaches may be useful when depicting adolescent food environments and related food behaviors. Further research is needed when evaluating story maps and how individuals can be trained to create their own maps.

## 1. Introduction

The built environment has been studied as a contributing factor to the increased exponential changes in the prevalence of obesity over the last fifty years [1,2,3,4]. The built environment encompasses all human-made aspects of our environments, and the food environment is one subset of the built environment. Specifically, the food environment is defined as places where individuals can acquire food items, such as restaurants, grocery stores, farmers’ markets, convenience stores, workplaces, schools, and home [5,6].

In the adolescent population, three primary food environments have been identified that influence food choice and consumption: school, the community, and home [7,8]. With rates of adolescent obesity steadily increasing in the last decade, researchers continue to investigate environmental and policy approaches to address the epidemic [9]. Evidence of the relationship between obesity and food environments, particularly for adolescents, is mixed, and methods used to analyze these environments typically focus on either neighborhood level data or perceptions of the environment [10,11,12,13].

Geographic information systems (GIS) have long been used to quantitatively assess food environments in terms of density or proximity to certain types of food outlets [5,14,15,16,17,18,19]. However, GIS professionals and social science researchers are now considering qualitative activity data, including interview quotes and pictures of a neighborhood taken from the perspective of community members, as helpful in explaining behaviors and experiences beyond what quantitative objective measurements are able to capture [20,21]. Another way that GIS data have been used with qualitative research is story mapping [21,22]. Typically used in community settings to allow stakeholders and community members to better understand their shared experiences, story maps embed photos, videos, comments, and other information in an online, interactive map. Story maps provide context and socially constructed information beyond objective assessments [23,24].

Research from Knigge and Cope has established grounded visualization as a methodology that can be used to incorporate qualitative data with GIS [25,26,27]. Based on grounded theory approaches, the process of grounded visualization is iterative in nature, exploring possibilities without a specific hypothesis a priori [22,28]. Use of this methodology can incorporate the knowledge and power of the community into the scientific process, often reducing the barriers of marginalized representations of underrepresented communities’ perspectives [22]. Walker and Hanchette used grounded visualization to establish a framework regarding neighborhood perspectives of a low-income population, displaced by local revitalization. They outlined this methodology in a three-pronged approach, which included mapping the studied neighborhood, conducting community member interviews, and using modified Photovoice methods termed “drive-by photography” [29].

Story mapping (with grounded visualization as a guiding methodology) may be an appropriate way to engage adolescents in action research and support them in working towards health promotion and behavior change outcomes [25,26,27]. The objective of this exploratory study was to better understand the perceptions of adolescents’ food environments, food behaviors, and choices using grounded visualization and story mapping. Similar to Walker and Hanchette’s three-pronged approach to grounded visualization, this paper used a four-pronged approach to advance scientific knowledge on how story mapping and use of qualitative GIS can be utilized to better understand the links between adolescent food environments and food choices [29].

## 2. Materials and Methods

In this convergent, multiphase, mixed methods study, data were collected from one high school in the southeastern United States from October 2016 to April 2017. The research team explored adolescent food environments, health behaviors, and demographic characteristics for a larger health-related study and then engaged a sub-population in focus groups and action research to provide further context. The methods are outlined based on a modified grounded-visualization, four-pronged approach resulting in a story map of information integrated from all stages of data collection and analyses [22,29]. All subjects gave their informed consent for inclusion before they participated in the study. The study was conducted in accordance with the Declaration of Helsinki, and the protocol was approved by the Ethics Committee of the University of Tennessee (UTK IRB-14-09366 B-XP) as well as the high school administration board.

### 2.1. Prong 1: Dietary behaviors data collection

Students from one high school were recruited via wellness class announcements, general school announcements, flyers, and face-to-face contact for six weeks (September and October 2016). T-shirts, pens, stadium cups, and other merchandise were provided to students to increase awareness of the larger research study, which encompassed this project. Students currently enrolled at the high school were eligible to participate if they had documented parental consent and provided assent. The survey was administered via an online platform and offered during class times and lunch periods. Of 565 students attending the school, 13.3% (*n* = 75) completed the online survey and were considered eligible. This aim of this prong was to understand the dietary behaviors and food environments of the school overall. Online survey components included dietary behaviors (fruit and vegetable (FV) intake, perception of support, and meal patterns), self-reported height and weight, and demographics [30,31]. Self-reported height and weight were used to calculate body mass index (BMI) [32,33]. ArcGIS online was used to develop multiple maps of the school, surrounding food environment, and census tracts of the county that students reside in based on data provided by the American Community Survey and census tracts [34]. Additionally, listings of potential food stores, convenience stores, grocery stores, and restaurants were identified surrounding the school environment with a three-mile buffer from Google maps with additional comparison maps and ground-truthing to verify [6,19,35].

### 2.2. Prong 2: Focus Groups

Individuals were recruited through high school wellness classes (*n* = 2 classes) to participate in focus groups using in-class announcements and flyers. Participants were deemed eligible to participate in Prong 2 if they met the previous eligibility criteria. Demographic data from the online surveys were linked to participants in the focus groups. The aim of Prong 2 was to glean information about how perceptions of adolescents’ food environments (from the adolescent viewpoint) related to food behaviors and the perceived factors that impact on those behaviors. Five focus groups were conducted, with approximately five to seven students in each group (*n* = 30 total participants). Participants were asked to elaborate on three food environments (school, community, and home), including facilitators and barriers to making desired food choices. A semi-structured interview guide was developed based on the socioecological model, social cognitive theory, and proposed adolescent food choice framework proposed by Story et al. [8,36,37,38,39,40,41]. Focus groups were audio recorded and transcribed verbatim.

### 2.3. Prong 3: Photovoice of Community and Home Environments

The research team invited all participants from the focus groups to participate in a modified, electronic Photovoice project to gain a more in-depth analysis of the community and home food environments [42]. Students were eligible to participate in Prong 3 if they met all previously stated criteria. Of 30 students who were asked to participate, 6 (20%) participated and submitted pictures online. Demographic data, including home addresses, were linked with the sub-sample. Participants were asked to take pictures of their community and home food environments during two different weeks using their cell phones; they were instructed to take pictures of anywhere they acquired food items, any foods they commonly eat, any meals, and depictions of the different types of food environments they encounter [43,44]. Instructions, a guide for ethical photography, and a written prompt were provided in the classroom [42,45,46,47]. The pictures were then uploaded by the participants to the online survey platform with an open space for the participant to comment on each picture [43,44].

In addition to the identification of major themes in pictures, travel activity patterns (identified in Prong 1 with mapping) were re-analyzed and associated with Photovoice pictures. Home food environments were mapped, and census data were used to assess the proximity and amount of food outlets near home. Additionally, the research team coded for the access and availability of food items around the home food environments and, looking along the travel activity patterns, estimated that of the school food environment.

### 2.4. Prong 4: Development of Story Map

The development of the story map began with data merging and integration based on a convergent, multiphase approach, outlined by Onwuegbuzie and Teddlie [48]. Baseline descriptive statistics were used to describe dietary behaviors and meal patterns and were performed using JMP version 14.0 to assist in quantitative data reduction [49]. Developed maps from food environments were also reviewed by a GIS analyst for common themes. Two researchers separately reviewed findings, noted common themes, and then discussed any discrepancies in themes. A modified Prong 1 data set was created based on these qualitative themes from the quantitative strand in Excel. Focus group analysis was conducted by the lead researcher, first with multiple rounds of first cycle coding (in vivo, process, and value), second cycle coding (focused), and code mapping to determine overall themes, and data organization was done on NVivo version 11.0 [50,51]. Photovoice and related comments were then coded separately, utilizing open coding (first cycle) and axial coding (second cycle) to develop separate themes. Major findings from all Prongs were merged to an Excel spreadsheet. A Prong 4 data set was created with themes from all comparisons (*n* = 11).

The Prong 4 themes were then used to develop a story map using ArcGIS Online [34]. As outlined in grounded visualization, researchers iteratively went back to previous maps and Prong data sets to ensure representation of themes and visualization was an accurate representation of participants’ experience in the story map [22]. No photographs taken during the modified Photovoice project were utilized in the story mapping application due to low resolution; to represent themes derived from coding Photovoice, stock photos were used. As a member check for validity, the story map was presented via email to the students who participated in Prongs 1–3 to ensure the map was reflective of their experiences [42,45,46,47]. Participants recommended changes in visual appeal, and these changes (*n* = 6) were made.

## 3. Results

### 3.1. Prong 1: Quantitative Dietary Behavior and Mapping

Participants in Prong 1 were white non-Hispanic (81.3%), Freshmen (74.7%), 14–15 years old (86.7%), and 54.1% were male. Twelve percent of the sample reported free or reduced lunch status; 29.3% chose not to answer or reported not knowing. The mean reported daily FV consumption was 2.71 (SD = 2.29) cups. Overall dietary patterns indicated that 48% consumed breakfast daily, and 54.7% consumed fast food at least once per week. Baseline demographics and dietary behaviors are further outlined in Table 1.

**Figure 1:**
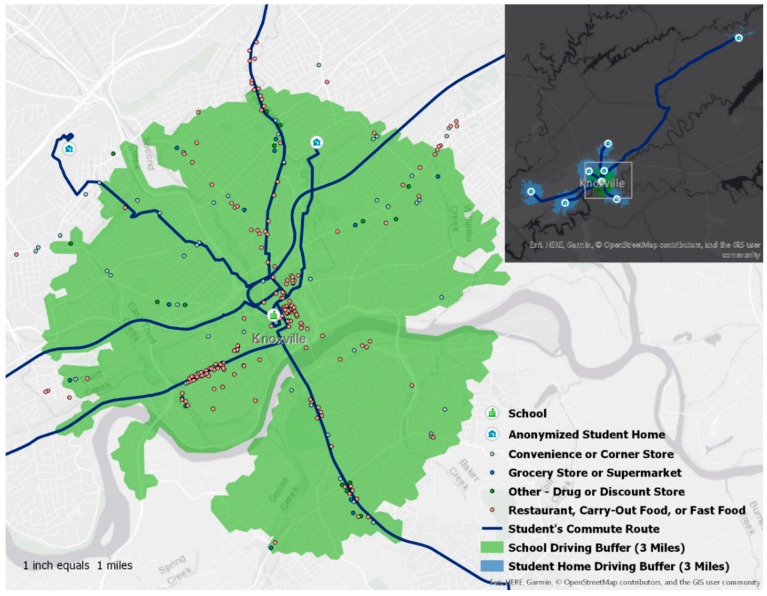
In-depth analysis of school food environment with buffer zone surrounding school highlighted (green).

Figure 1 depicts the school food environment with the sub-sample of participants’ (*n* = 6) community food environments highlighted in blue with potential travel activity patterns (based on population density and major roadways) outlined in red. Of 262 food sources (grocery stores, convenience stores, drug stores, discount stores, and restaurants) identified in the school’s 3-mile buffer zone, 154 (58.8%) were restaurants, primarily fast food or quick service. One important thing of interest concerning the sub-sample was that the participants resided in all areas of the county, including one who lived outside of the county, commuting over one hour each way per day.

### 3.2. Prong 2: Focus Groups

The Prong 2 sample was similar demographically to Prong 1; 80% reported being white non-Hispanic, Freshmen (86.7%), and 14 years old (73.3%). Three overarching themes emerged and were apparent in all three food environments: Convenience (use of grab-and-go meal and snack items), irregularity (irregular meal patterns, particularly with differences on week and weekends), and control (independence of food choices and meals). Overall, youth reported issues related to convenience, lack of time due to extracurricular activities, and busy schedules that limit family meals as factors that increase fast food consumption and promote an unhealthy community and home food environment. Similar to current literature, convenience was of utmost importance to participants in this sample, citing it as a common reason for consuming fast food and snack items.

Two novel findings in this prong were related to use of technology for meal planning and influence of independent travel activity via personal vehicle on food behaviors. Youth also identified use of technology (including phone applications) in meal preparation and meal planning, particularly when used in conjunction with other family members, as ways to be more involved in the home food environment. Specifically, the youth identified that using group texting and applications were a way for them to contribute to the family shopping list. Online grocery ordering done by youth and their families as well as participation in meal subscription boxes were also notable characteristics of engagement in technology to participate in meal planning and preparation activities. Participants in this sample did not have driver’s licenses and reported having a driver’s license was a critical component for increased independent food acquisition for high school students. Thus, participants without driver’s licenses reported that food acquisition was limited to times when they were traveling with parents or friends and acknowledged that independent travel activity may alter community food environment exposure.

### 3.3. Prong 3: Modified Photovoice Sub-Sample

The Prong 3 sample reported being white non-Hispanic (57.1%), with the remaining participants reporting being biracial and/or Hispanic, all Freshmen students (100%), and 14 years old (71.4%). 57.1% of the sample reported being male. Similar to Prong 2 findings, convenience was an overarching theme of Photovoice analysis. Snack food items were prominent in the home food environment, with 26.2% of photographs including snack items (as identified by participants in the comments). Pictures of snack cabinets, fruit bowls, and stocked refrigerators were common for the home food environment with the sub-sample. Family meals were also frequently depicted, with some participants noting special holiday meals and theme nights as reasons for eating together. 

In the community food environment photographs, a divergence of snack food options was depicted at home versus non-home settings. Gas stations and convenience stores were reported as sources of high-fat, high-sugar foods and beverages when not at home, but fresh fruits, vegetables, and whole grain options were offered at home more frequently. Convenience was also depicted in both community and home food environments through photographs of fast food outlets and bringing quick meals home. Participants frequently took photographs of food outlets from a vehicle while riding with another person.

### 3.4. Prong 4: Development of Story Map to Describe Adolescent Food Environments

**Figure 2:**
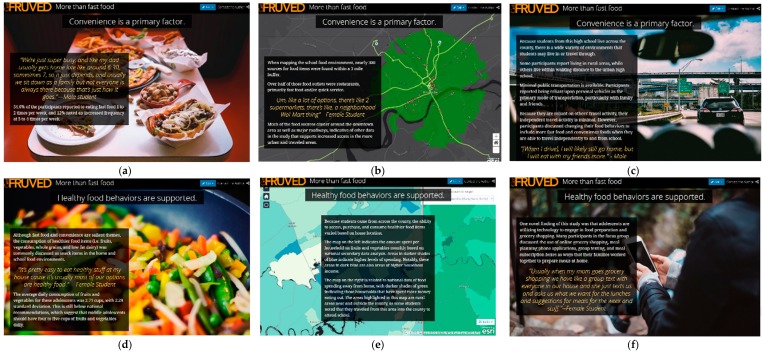
Pictorial depiction of online, interactive story map as follows: (**a**) Start of convenience section where fast food is depicted for family meals and embedded quote from focus groups; (**b**) next convenience section where school food environment with buffer zone and identified food outlets are shown; (**c**) transportation shown with narrative regarding dependent travel activity and embedded quotes from focus groups; (**d**) the next section depicting support of healthy behaviors starts with cooking skills; (**e**) mapping of county region from Prong 1; (**f**) use of technology with meal planning and preparation shown.

The new data set derived from analysis included seven overall themes (indicated in Table 2) with 1 to 2 sub-themes fitting under most categories. Based on integration of Prongs 1–3, some new themes that were generated in Prong 4 included cooking skills, FV intake, family support of healthy food behaviors, and limited food access for some. Figure 2 is a pictorial description of the map. A detailed description of the new themes and how they relate to the story map follow below (in Table 2).

### 3.5. Detailed Description of Story Map

The story map exists on ArcGIS Online, a cloud-based system that allows anyone with the hyperlink to visit. Interaction with the story map is often done with scrolling and zooming capabilities. It is important to note that the maps are the central theme in a story map and should be considered prior to adding photos or words. On these first slides, the location and description of the sample are shown to assist in providing context to the adolescents’ perspectives from this sample. This section includes demographics of the overall sample from Prong 1, the purpose of the study, a map describing the geographical location, and specifically noted the driving status of this sample. A regional map of the sample’s location is also included. In the next block of slides, the focus is placed on convenience, as this was a prominent theme in all prongs, and quotes from the focus groups are used to illustrate this concept. For example, a picture of the family meal with fast food options is depicted with a focus group quote stating, “We’re just super busy, and like my dad gets home late, like around 6:30 or sometimes 7, so it just depends, and we usually sit down as a family but not everyone is always there because that’s just how it goes.” In this section, two students from the Photovoice sub-sample were chosen to illustrate differences in rural and non-rural individuals from this area. The home environment in the rural area depicts limited access to gas stations and/or food outlets with none noted. Compared to the rural area, the other participant lives in a suburban environment, with access to multiple grocery stores, restaurants, and outlets for food acquisition. This section also presents the importance of transportation and displays the school food environment that all participants share. The green zone is a 3-mile radius surrounding the school, with 300 food sources identified in this area (depicted in Figure 1 as well). Major roadways are highlighted in light green, leading to the sub-samples’ home addresses. Wide variance exists between the sub-sample and their home food environments and travel activity patterns, despite having a common school environment. The last section focuses on support of healthy behaviors, addressing the perceived differences in healthier food items being available in the home as well as assistance with meal preparation, both directly and indirectly with technology. Based on the Prong 4 data, support for healthy behaviors from family and peers was a critical component in the youth’s behaviors. Thus, the discussion surrounding family meals and assistance with cooking was also dependent upon if parents or caregivers expected participation from the youth and if busy schedules limited them. The technology component was highlighted by one focus group quote from a female participant, stating, “Usually when my mom goes grocery shopping we have like a group text with everyone in our house and she just texts us and asks us what we want for the lunches and suggestions for meals for the week…”

## 4. Discussion

Much of the data derived from both qualitative and quantitative strands of data were reflective of current literature regarding adolescent food environments, including issues related to convenience, use of fast food restaurants as a food source, and busy schedules that limit family meals [7,8,52,53]. However, novel findings for nutrition literature related to the use of technology and travel activity were also common themes from all prongs. New themes based on the analysis of the integrated data set that were not specifically identified with either qualitative or quantitative analysis included the importance of cooking skills as well as familial and peer support for healthy behaviors. Some of these differences may exist due to the unique nature of the middle adolescent period in which independence is emerging, while also peer and family support are still prominent.

Data integration from the quantitative and qualitative strands was mostly convergent, but there were some notable divergences as well. Support of healthy behaviors, particularly from parental influence in the home food environment, was another prominent theme in Prong 4 with divergent data. Although participants reported increased availability of healthier food items due to parental food acquisition and positive role modeling making it easier to eat healthier food items at home, some participants in Prong 2 noted that parents often provide negative role modeling by providing high-fat, high-sugar items in the home that are tempting, particularly when parents are consuming them frequently. These findings support previous research conducted by Anderson Steeves et al. [54].

Story mapping has been commonly used in community settings to spark conversation surrounding pertinent issues. However, the development and use of story mapping for health promotion and related behavior change is an underdeveloped area in peer-reviewed publications [55,56]. Thus, a better understanding of ways to develop the map in the web-based application as well as effective, evidence-based methods for presenting back to the community with evaluation is the necessary next steps in the literature. Some literature indicates appropriate teaching methods of story map development to adult learners and ways for community members to create their own story maps [23,57,58]. Further engagement in the research with participants directly developing the story map from training provided by researchers may also be a mechanism for community action and behavior change.

Many aspects of the study are unique. First, the use of grounded visualization and critical GIS methodology to incorporate both perceptions and observations of food environments is a new, developing approach, but one that addresses previous gaps in the literature. Based on grounded visualization with an embedded, mixed methods framework, data analysis and interpretation were an iterative process that provided rich context beyond quantitative data alone. Additionally, because all food consumption is important when conveying the participant experience, the research team refrained from coding Photovoice food items and meals as “healthy” or “unhealthy”. These categorical terms are subjective in nature, and the objective of the project was to accurately reflect adolescent food environments from this sample’s perspective using story mapping and qualitative GIS approaches. The research team simply considered what environments and context related to acquisition or consumption of healthier food items when doing qualitative data analysis to decrease this known bias.

Although grounded theory is well developed and understood, the use of qualitative theory in GIS and spatial analysis is fairly new, particularly in nutrition and health promotion research [22,23,28,29,59]. However, many of the gaps previously identified in nutrition and food environment research have been focused on combining individual behavior and perceptions with environmental aspects, consistent with social cognitive and socioecological model theories [37,38,60]. The use of critical GIS and grounded visualization helps to bridge that gap, despite its novelty [22]. However, sample size has been difficult to determine with this methodology [22]. Typical geospatial analyses rely on large amounts of data at a population level. However, the focus groups and other qualitative data are often done with smaller samples, allowing for decreased spatial analysis in mapping software when incorporating the two types of data [22,59]. Appropriate data collection methods and ways to evaluate the use of story maps have limited evidence in peer-reviewed publications.

One limitation of this study is the use of convenience sampling. This sampling framework used across all methods of data collection created a sample that may not be representative of all adolescents, nor the school overall. Additionally, participants who continued as part of the sub-sample in Prong 3 may not be the most representative of the entire sample because those who continued participation may have an increased interest in discussing health-related issues or engaging in health promotion efforts. Thus, the story map that we developed may be unique to those youth who are more interested in health and nutrition, and later community engagement with the maps may be altered based on this perspective. Also notable is the low sample size as the prongs in the study progress, and the sub-sample engaged in the modified Photovoice procedures was six. Wang et al. recommended an optimal Photovoice sample size of 7 to 10 participants, and Walker and Hanchette used five participants for their interview and drive-by photography approach to develop a narrative story map [29,42]. Another limitation was the absence of an interview with the sub-sample who completed the modified Photovoice. Conducting an interview with the adolescents and allowing the sub-sample to choose photographs to be included in the story map aligns more closely with typical Photovoice methods and it was not possible for it to be conducted in this study. To mitigate this slightly, the research team allowed participants to provide comments when submitting pictures and following the creation of the story map.

## 5. Conclusions

Use of grounded visualization and story mapping may be useful tools when evaluating adolescent food environments and related food behaviors. Future research should evaluate the effects of developed story maps when presenting back to the population of interest, particularly for behavior change. Additional research needs to be conducted on the use of grounded visualization with other populations and their food environments, as well as effective ways to develop and evaluate this data visualization tool.

## Figures and Tables

**Table 1:** Baseline characteristics from Prong 1 sample (*n* = 75). FV = Fruit and vegetable, BMI = Body mass index.

Characteristic	Count (%) or Mean ± SD
**Age (years)**	
13	1 (1.3)
14	42 (56)
15	23 (30.7)
16	7 (9.3)
17	2 (2.7)
**Year in School**	
Freshmen	56 (74.7)
Sophomore	13 (17.3)
Junior	5 (6.7)
Senior	1 (1.3)
**Gender (*n* = 74)**	
Male	40 (54.1)
Female	34 (45.9)
**Race**	
White only (non-Hispanic)	61 (81.3)
Black only (non-Hispanic)	4 (5.3)
Other (including biracial and Hispanic/Latino)	10 (13.4)
**Free/Reduced Lunch**	9 (12)
**FV Intake (cups)**	2.71 ± 2.29
**BMI (%)**	21.71 ± 4.08
**Vegetarian**	7 ± 8.1

**Table 2:** Data integration from all four prongs to display development of story map themes.

Comparison of Information from Prongs 1–3 to Develop New Prong 4 Themes			
Prong 4	Prong 1	Prong 2	Prong 3
**Convenience**	• Places of food acquisition centrally located in more urban areas and near major roadways • Limited food access for some in more rural areas	• Busy schedule for both adolescents and parents as a reason for convenience foods • Decreased price compared to healthier options	• Grab-and-go snacks • Quick service meals for family meals • Meals on go while heading to next place
**Fruit and vegetable (FV) intake**	• 1.87 cups daily (Range: 0.25 to 13 cups)	• Increased availability of FV at home	• Fresh fruit and vegetables depicted in home and taking in school lunch
**Fast food**	• 32% reported never consuming in last week • 54.6% reported 1–2 times per week consumption • Mainly fast food and quick service restaurants in three-mile radius	• Increased availability of fast food	• Fast food outlets and quick service meals at home
**Support of healthy behaviors**	• 66.6% reported friends think it is “somewhat” or “very much” important to be healthy	• Parents provide positive role modeling for healthy eating • Parents provide access to healthy foods • Parents sometimes are negative role models for healthy eating	• Access to FV in home provided by parents • Snacks provided by parents are healthier items • Family meals at dinner table
**Travel activity**	• Limited public transportation options across county • Must use personal vehicle to access	• No drivers’ license • Relied on family and friends	• Community pictures while riding in car with family member
**Cooking skills**	N/A	• Parents cook frequently with adolescents’ help • Starts preparing dinner for family at times • Satisfaction in being able to assist family with cooking • Prepares meals for self frequently	• Meal preparation
**Technology**	N/A	• Use of phone applications, group texting, food subscription boxes, online food shopping to acquire food items • Family uses online recipes frequently	• Pictures of meals and food from social media outlets and internet influenced food choices

## Supplementary Materials

The following are available online at https://myutk.maps.arcgis.com/apps/Cascade/index.html?appid=37a52a89246c4f42a234496d117e0a7f.
